# Genetic Risk Score Modelling for Disease Progression in New-Onset Type 1 Diabetes Patients: Increased Genetic Load of Islet-Expressed and Cytokine-Regulated Candidate Genes Predicts Poorer Glycemic Control

**DOI:** 10.1155/2016/9570424

**Published:** 2016-01-20

**Authors:** Caroline A. Brorsson, Lotte B. Nielsen, Marie Louise Andersen, Simranjeet Kaur, Regine Bergholdt, Lars Hansen, Henrik B. Mortensen, Flemming Pociot, Joachim Størling, Hvidoere Study Group on Childhood Diabetes

**Affiliations:** ^1^Copenhagen Diabetes Research Center (CPH-DIRECT), Department of Pediatrics E, University Hospital Herlev, 2730 Herlev, Denmark; ^2^Novo Nordisk A/S, 2760 Måløv, Denmark; ^3^DECCP, Clinique Pédiatrique, CH de Luxembourg, 4 rue Barblé, 1210 Luxembourg, Luxembourg

## Abstract

Genome-wide association studies (GWAS) have identified over 40 type 1 diabetes risk loci. The clinical impact of these loci on *β*-cell function during disease progression is unknown. We aimed at testing whether a genetic risk score could predict glycemic control and residual *β*-cell function in type 1 diabetes (T1D). As gene expression may represent an intermediate phenotype between genetic variation and disease, we hypothesized that genes within T1D loci which are expressed in islets and transcriptionally regulated by proinflammatory cytokines would be the best predictors of disease progression. Two-thirds of 46 GWAS candidate genes examined were expressed in human islets, and 11 of these significantly changed expression levels following exposure to proinflammatory cytokines (IL-1*β* + IFN*γ* + TNF*α*) for 48 h. Using the GWAS single nucleotide polymorphisms (SNPs) from each locus, we constructed a genetic risk score based on the cumulative number of risk alleles carried in children with newly diagnosed T1D. With each additional risk allele carried, HbA1c levels increased significantly within first year after diagnosis. Network and gene ontology (GO) analyses revealed that several of the 11 candidate genes have overlapping biological functions and interact in a common network. Our results may help predict disease progression in newly diagnosed children with T1D which can be exploited for optimizing treatment.

## 1. Introduction

In type 1 diabetes (T1D) the pancreatic *β*-cells are destroyed by the immune system in a process involving the proinflammatory cytokines interleukin-1-*β* (IL-1*β*), interferon-*γ* (IFN*γ*), and tumor necrosis factor-*α* (TNF*α*) released from antigen-presenting cells and T-cells [1, 2]. Genome-wide association scans (GWAS) have identified more than 40 genomic regions that are associated with T1D risk [3] (http://www.t1dbase.org). Many of the GWAS candidate genes have annotated immune-cell functions and most of the genetic risk variants have therefore been suggested to modulate immune-regulatory pathways [4, 5]. However, recent studies have highlighted that a significant proportion of the candidate genes are also expressed in human islets suggesting functional effects in *β*-cells [6–8] and possibly involvement in inflammation- and immune-mediated *β*-cell killing mechanisms thereby potentially affecting disease progression after clinical onset [9]. As most variants identified through GWAS contribute to only modest effects to disease risk, it is likely that a combination of variants will better capture effects of clinical relevance. In T1D, very few studies have analyzed the impact of multiple variants on disease prediction and progression [10–12], although candidate gene-focused studies have demonstrated association with parameters of disease progression [13–16].

In the current study, we aimed at investigating whether a combined genetic risk score of T1D risk variants can predict glycemic control and residual *β*-cell function as assessed by HbA1c and insulin dose-adjusted HbA1c (IDAA1c) during disease progression in children with newly diagnosed T1D. We exclusively included SNPs for candidate genes expressed and transcriptionally regulated by cytokines in the target tissue of T1D, that is, human islets, as we hypothesized that these qualify as the most directly involved predictors.

## 2. Research Design and Methods

### 2.1. Expression Profiling of Candidate Genes in Human Islets

Human pancreatic islet preparations from nine nondiabetic donors (aged 8–57 years; 6 males and 3 females) were obtained from a multicenter European Union-supported program on *β*-cell transplantation in diabetes. None had classical T1D-associated HLA-DR risk genotypes. The program was approved by central and local ethical committees. Islet preparation, cytokine stimulation (5000 U/mL TNF*α* + 750 U/mL IFN*γ* + 75 U/mL IL-1*β* for 48 h), and RNA extraction have been described previously [17]. Relative gene expression of candidate genes was evaluated by TaqMan assays using the Low Density Array system on TaqMan 7900HT (Applied Biosystems). Target gene expression was normalized to the geometric mean of three housekeeping genes (*GAPDH*,* 18S-RNA*, and* PPIA*) and evaluated using the delta-delta Ct method [18]. One of the identified genes (*IL10*) whose expression was modulated following cytokine treatment was only detected in three of the human islet preparations. Genes with Ct values < 37 were considered as expressed.

### 2.2. Study Populations from the Hvidoere Study Group (HSG) on Childhood Diabetes

The study population was collected through HSG and is described in [19]. The cohort included in total 257 children (126 girls and 131 boys). Eighty-four percent of the patients were white Caucasian, and age at clinical diagnosis was 9.1 ± 3.7 years (mean ± SEM), BMI 16.5 ± 3.2 kg/m^2^, and HbA1c 11.2 ± 2.1% at the time of diagnosis. DKA (HCO3 ≤ 15 mmol/L and/or pH ≤ 7.30) was present in 20.7% of the cases at the time of diagnosis. Exclusion criteria were suspected non-T1D (type 2 diabetes, maturity-onset diabetes of the young (MODY), or secondary diabetes), decline of enrolment into the study by patients or parents, and patients initially treated outside of the centers for more than 5 days. The diagnosis of T1D was according to the World Health Organization criteria. The study was performed according to the criteria of the Helsinki II Declaration and was approved by the local ethic committee in each center. All patients, their parents, or guardians gave informed consent. In the current study, patients with missing values for genotyping and clinical outcome measures were excluded leaving a total of 182 patients with complete genotype profile and clinical characterization.

#### 2.2.1. HbA1c and IDAA1c (Insulin Dose-Adjusted HbA1c)

HbA1c was analyzed centrally by ion-exchange high-performance liquid chromatography at onset and 1, 3, 6, 9, and 12 months after diagnosis. IDAA1c is defined as actual HbA1c + (4 × insulin dose (U/Kg/24 h)). A calculated IDAA1c ≤ 9 corresponds to an estimated maximal C-peptide level above 300 pmol/L and has been used to define clinical remission [20].

### 2.3. Genotyping

Genotyping of rs2290400/*GSDMB*, rs2327832/*TNFAIP3*, rs4948088/*COBL*, rs7202877/*CTRB1*, rs7804356/*SKAP2*, rs1990760/*IFIH1*, rs3184504/*SH2B3*, rs6897932/*IL7R*, rs3024505/*IL10*, rs3825932/*CTSH*, and rs689/*INS* was done using the KASPar system (KBioscience, Hoddesdon, UK). Typing of the HLA-class II DRB1 locus was performed by direct sequencing of exon 2 of* DRB1* according to Immuno Histocompatibility Working Group. The HLA risk groups were defined as high risk (*DRB1* 03/04, 04/04), moderate risk (*DRB1* 03/03, 04/08), and low risk (all other* DRB1* genotype combinations).

### 2.4. Gene Ontology Terms and Network Construction

We used PANTHER [21] to perform functional annotation of the 11 input candidate genes. The enrichment for gene ontology (GO) terms in the biological process category was identified based on binomial test. The human genome was used as the reference list. To construct protein networks on the 11 input candidate genes, the STRING network tool was used. STRING is a database of known and predicted protein interaction data from multiple sources including experiments, coexpression, and text mining. In total, STRING covers nearly 10,000,000 proteins from over 2,000 organisms (http://string-db.org). Network was built with a medium confidence score (0.400) and up to 10 interactors.

### 2.5. Statistical Analysis

A genetic risk score was calculated for each individual based on the cumulative number of risk alleles carried for the 11 SNPs and was used as a continuous variable to test for association with IDAA1c and HbA1c levels at 1, 3, 6, 9, and 12 months after T1D onset in linear regression models. The assigned risk alleles for* CTSH* and* SKAP2* were opposite compared to risk of T1D due to regression analyses from individual SNP models. Regression models were adjusted for the covariates sex, age group (0–5, 5–10, and >10 years at diagnosis), and HLA risk groups. Forward stepwise regression models were selected from all SNPs and covariates. A *p* value below 0.05 was considered statistically significant. All statistical analyses were performed in SAS version 9.2.

## 3. Results

### 3.1. Cytokine-Induced Gene Expression in Human Islets

**Figure 1:**
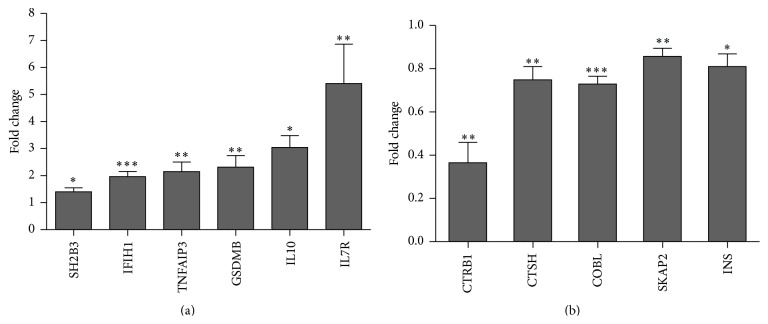
Cytokine-regulated candidate genes in human islets. Isolated human islets were left untreated or exposed to cytokines (IL-1*β* + IFN*γ* + TNF*α*) for 48 h. Gene expression of candidate genes was determined by real-time PCR. Target gene expression was normalized to the geometric mean of three housekeeping genes. (a) Genes upregulated in response to cytokine treatment. (b) Genes downregulated in response to cytokine treatment. Data are means ± SEM of *n* = 8-9, except for IL10 (*n* = 3). ^*∗*^ *p* < 0.05, ^*∗∗*^ *p* < 0.01, and ^*∗∗∗*^ *p* < 0.001.

Gene expression may represent an intermediate phenotype between genetic variation and disease. We therefore first evaluated the expression of established/pinpointed T1D GWAS candidate genes in human islets left untreated or exposed to a combination of proinflammatory cytokines (IL-1*β* + IFN*γ* + TNF*α*) for 48 hrs to mimic disease. We found 31 out of 46 tested genes to be expressed. Of these, 11 significantly changed their expression level following cytokine treatment (*p* < 0.05) (Table 1). Six candidate genes were upregulated by cytokines,* TNFAIP3*,* IFIH1*,* GSDMB*,* IL7R*,* IL10,* and* SH2B3*, whereas 5 genes were downregulated,* COBL*,* CTRB1*,* CTSH*,* SKAP2,* and* INS *(Figure 1). Comparable expression profiles of these genes were observed in a recently published human islet dataset [6].

### 3.2. Genetic Risk Score Modelling of Glycemic Control and *β*-Cell Function

**Figure 2:**
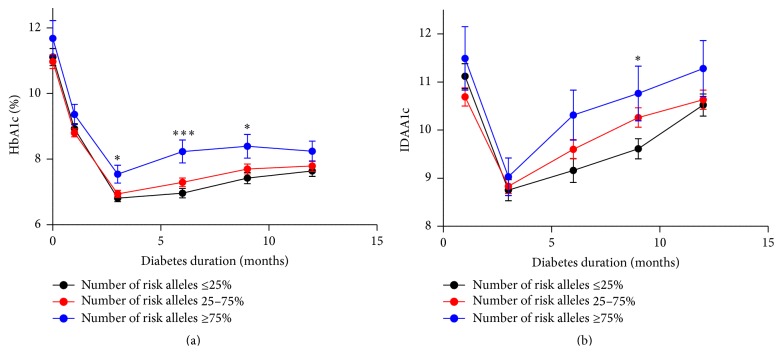
Correlation between HbA1c and IDAA1c levels and risk allele numbers. HbA1c (a) and IDAA1c (b) in carriers with <25% (*n* = 65), 25–75% (*n* = 96), or >75% (*n* = 21) risk alleles at 1, 3, 6, 9, and 12 months following disease onset. Data are means ± SEM, ^*∗*^ *p* < 0.05, ^*∗∗∗*^ *p* < 0.001.

A genetic risk score model was constructed from the GWAS-identified SNPs linked to the 11 genes identified above to investigate the cumulative effect of T1D-associated risk alleles on disease progression in new-onset T1D children. The risk allele distribution is described in Supplementary Table 1 in Supplementary Material available online at http://dx.doi.org/10.1155/2016/9570424. HbA1c and IDAA1c (a surrogate marker for *β*-cell function [20]) levels were increased in carriers with risk allele numbers at and above the 75th percentile (corresponding to minimum 15 risk alleles) during disease progression (HbA1c: 3, 6, and 9 months after disease onset, *p* = 0.04, *p* = 0.0004, and *p* = 0.03, resp.; and IDAA1c: 9 months, *p* = 0.04) (Figures 2(a) and 2(b)).

We then performed a multiple linear regression analysis adjusted for age, sex, and HLA risk groups and found significantly increased HbA1c and IDAA1c levels with increasing genetic risk score (GRS) from 3–12 months following T1D onset (Table 2). The validity of including GRS in the regression analysis was tested by comparing the variance explained by the model (*R* ^2^). This clearly showed that including GRS as explanatory factor improved the model (Supplementary Table 2). These findings suggest that residual *β*-cell function declines faster following diagnosis in patients carrying increased genetic load of islet-expressed and cytokine-regulated candidate genes.

### 3.3. Network and GO Analyses of Candidate Genes

**Figure 3:**
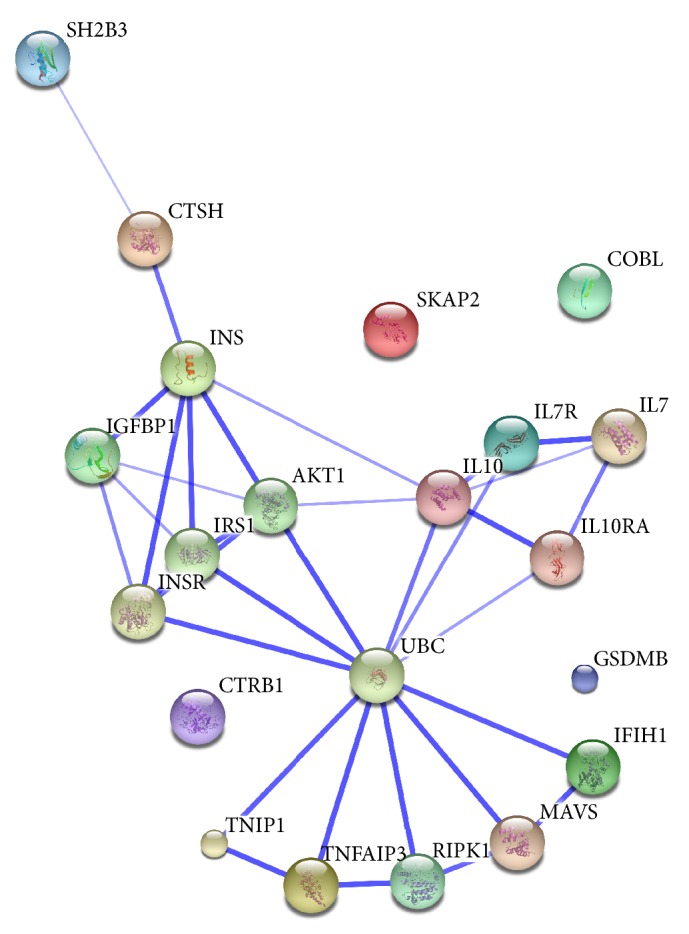
Protein interaction network of the 11 genes. The network was constructed using the STRING tool (http://string-db.org) and the 11 candidate genes as input. The width of the interactions depends on the confidence score to each association in STRING.

We next asked if any of the 11 candidate genes may interact with each other in a functional protein network which could explain their cumulative effects on disease progression. This was evaluated by the STRING network tool which constructed a network that contained 7 out of the 11 genes (Figure 3). Consistent with this, the functional annotation of these candidate genes based on GO analyses revealed significantly enriched GO terms in biological processes category (Table 3). The 11 candidate genes were found enriched for various immune-mediated processes including regulation of immune response (*p* = 0.0008) and immune system process (*p* = 0.01). These findings support that several of the 11 candidate genes act in common networks and pathways to affect disease risk and progression.

## 4. Discussion

Recent GWAS have identified a large number of loci affecting T1D risk [3]. In this study, we investigated the clinical relevance of a genetic risk score on markers of disease progression. An increased genetic risk score associated with increasing HbA1c and IDAA1c levels the first year after disease onset, indicating that a higher genetic load of islet-expressed candidate genes predicts poorer glycemic control and residual *β*-cell function, respectively. One additional risk allele resulted in a 0.15% point increase in HbA1c after 12 months and a corresponding 0.19% increase in IDAA1c corresponding to a calculated 4% lower stimulated C-peptide [20]. Our cumulative genetic risk score assumes that each risk variant contributes with equal effects to the traits, which probably does not reflect the true underlying biology. An alternative approach would be to weight each variant by published effect sizes for T1D risk, which has been done in type 2 diabetes [22, 23]. We chose the unweighted cumulative score because disease risk and disease progression are different outcomes, which will likely not have identical effect sizes. This is underlined by our previous observation that there is no statistically significant association between HLA risk and T1D progression [19]. This is also the reason why we did not include HLA risk genes in the risk score model.

The observed poorer glycemic control associated with higher genetic load might prove to be a valuable tool for prediction of disease progression. This should, however, be validated in independent cohorts. An advantage of our study is that the inclusion of variants in the genetic risk score was based on prior “biological” knowledge, as we strictly focused on islet-expressed and cytokine-regulated candidate genes. Because regulated gene expression is often highly dynamic due to positive and negative feedback mechanisms, that is, the expression of a specific gene might be increased at one time point but decreased in another and vice versa, we did not take into account in the risk score model whether genes were up- or downregulated by cytokines but simply focused on the fact that their expression level changed as we considered this most important. We may have missed genes that changed expression at different time points compared to those examined at the 48 hrs, and a more detailed time-course study in human islets would likely have allowed a greater number of SNPs to be included in the risk score and thus provide even more accurate predictions.

Interestingly, we found that 7 of the 11 investigated genes interact in a protein network and several of the genes also shared GO terms suggesting that they affect the same biological mechanisms within the *β*-cells. We hypothesize that the genes are modulating *β*-cell function in terms of insulin secretion and/or the regenerative capacity and/or regulate the vulnerability of the *β*-cell to immune-mediated destruction. Indeed for some of the candidate genes, functional studies in *β*-cells have been performed. Hence, we recently demonstrated that CTSH regulates insulin gene transcription and secretion and also has antiapoptotic properties in *β*-cells [16]. Similarly, A20, the protein name of the gene product encoded by* TNFAIP3*, is an antiapoptotic protein that inhibits apoptosis induced by cytokines by blocking activation of the transcription factor NF*κ*B [24]. In conclusion, a cumulative genetic risk score comprising variants from 11 islet-expressed candidate genes predicted significantly poorer glycemic control and *β*-cell function during disease progression in new-onset T1D children. This knowledge might be useful to better predict disease progression after diagnosis with T1D.

## Supplementary Material

Supplementary Table 1: The risk allele distribution of the 11 T1D candidate genes.
Supplementary Table 2.: Variance explained by regression models for HbA1c and IDAA1c with and without genetic risk score (GRS).

## Figures and Tables

**Table 1:** The 46 T1D candidate genes tested for expression and cytokine regulation in human islets.

Region	GWAS SNP	Locus	Gene tested
1p13.2	rs2476601	*PTPN22*	*PTPN22*
1p31.3	rs2269241	*PGM1*	*PGM1*
1q31.2	rs2816316	*RGS1*	*RGS1*
1q32.1	**rs3024505**	*IL10*	***IL10***
2p25.1	rs1534422	*(Gene desert)*	
2q12.1	rs917997	*IL18RAP*	*IL18RAP*
2q24.2	**rs1990760**	*IFIH1*	***IFIH1***
2q33.2	rs3087243	*CTLA4*	*CTLA4*
3p21.31	rs11711054	*CCR5*	*CCR5*, *CCR3*
4p15.2	rs10517086	*(Gene desert)*	
4q27	rs4505848	*IL2*	*IL2*, *IL21*, *ADAD1*
5q13.2	**rs6897932**	*IL7R*	***IL7R***
6p21.32	rs9268645	*MHC*	Not included
6q15	rs11755527	*BACH2*	*BACH2*
6q22.32	rs9388489	*CENPW (C6orf173)*	Not tested
6q23.3	**rs2327832**	*TNFAIP3*	***TNFAIP3***
6q25.3	rs1738074	*TAGAP*	*TAGAP*
7p15.2	**rs7804356**	*SKAP2*	***SKAP2***
7p12.1	**rs4948088**	*COBL*	***COBL***
9p24.2	rs7020673	*GLIS3*	*GLIS3*
10p15.1	rs12251307	*IL2RA*	*IL2RA*
10p15.1	rs11258747	*PRKCQ*	*PRKCQ*
10q23.31	rs10509540	*RNLS*	*RNLS*
11p15.5	rs7111341, **rs689**	*INS*	***INS***
12p13	rs4763879	*CD69*	*CD69*
12q13.2	rs2292239	*ERBB3*	*ERBB3*
12q24.12	**rs3184504**	*SH2B3*	***SH2B3***
14q24.1	rs1465788	*C14orf181*	*C14orf181*
14q32.2	rs4900384	*(0*; *gene desert)*	
15q25.1	**rs3825932**	*CTSH*	***CTSH***
16p13.13	rs12708716	*CLEC16A*	*CLEC16A*, *PRM3*, *TNP2*
16p12.3	rs12444268	*UMOD*	*UMOD*
16p11.2	rs4788084	*IL27 (NUPR1)*	*IL27*, *NUPR1*
16q23.1	**rs7202877**	*CTRB1*	***CTRB2***; ***CTRB1***
17p13.1	rs16956936	*DNAH2*	*DNAH2*
17q12	**rs2290400**	*ORMDL3 (GSDMB)*	*ORMDL3*, ***GSDMB***
17q21.2	rs7221109	*SMARCE1*	*SMARCE1*
18p11.21	rs1893217	*PTPN2*	*PTPN2*
18q22.2	rs763361	*CD226*	*CD226*
19q13.32	rs425105	*PRKD2*	*PRKD2*
20p13	rs2281808	*SIRPG*	*SIRPG*
21q22.3	rs11203203	*UBASH3A*	Not tested
22q12.2	rs5753037	*HORMAD2*	*HORMAD2*
22q13.1	rs229541	*C1QTNF6*	*C1QTNF6*
Xq28	rs2664170	*GAB3*	*GAB3*

The genes that were transcriptionally regulated by cytokines in human islets are highlighted in bold, as are the corresponding risk SNPs included in the genetic risk score analysis.

**Table 2:** Impact on HbA1c and IDAA1c by increasing genetic risk score.

Time after onset	Increase in HbA1c (%) per additional risk allele (SE)	*p* value	Increase in IDAA1c per additional risk allele (SE)	*p* value
1 month	0.09	0.06	—	NS
3 months	0.11 (0.04)	0.009	—	NS
6 months	0.17 (0.05)	0.0006	0.16 (0.08)	0.04
9 months	0.14 (0.05)	0.01	0.19 (0.07)	0.01
12 months	0.15 (0.06)	0.008	0.19 (0.08)	0.02

The influence of increasing risk allele number on HbA1c and IDAA1c analyzed by genetic risk score generated from 11 qualified T1D genes in linear regression analysis adjusted for age, sex, and HLA risk groups. Data are presented as increase in HbA1c (%) and IDAA1c per additional risk allele during the first year after diagnosis in 182 children with new onset T1D.

**Table 3:** The gene ontology terms of the 11 T1D candidate genes.

GO biological process	Reference	Count	Genes	Expected	*p* value
Regulation of immune response	930	7	IFIH1, INS, TNFAIP3, IL7R, SKAP2, CTSH, IL10	0.49	0.0008

Negative regulation of immune response	116	4	INS, TNFAIP3, IL7R, IL10	0.06	0.002

Positive regulation of multicellular organismal process	1357	7	IFIH1, INS, TNFAIP3, COBL, IL7R, CTSH, IL10	0.72	0.01

Negative regulation of type I interferon production	43	3	IFIH1, TNFAIP3, IL10	0.02	0.01

Immune system process	2163	8	IFIH1, SH2B3, INS, TNFAIP3, IL7R, SKAP2, CTSH, IL10	1.14	0.01

Regulation of immune system process	1473	7	IFIH1, INS, TNFAIP3, IL7R, SKAP2, CTSH, IL10	0.78	0.02

Negative regulation of chronic inflammatory response	5	2	TNFAIP3, IL10	0	0.02

The enriched gene ontology (GO) terms in the biological process category are listed for the 11 candidate genes. The GO terms are followed by number of genes having the enriched term in the reference list (Reference), number of genes in the input list having the enriched term (Count), gene names for the genes listed in Count, and Bonferroni-corrected *p* values.
