# Supplementary material for: Genetic Risk Score Modelling for Disease Progression in New-Onset Type 1 Diabetes Patients: Increased Genetic Load of Islet-Expressed and Cytokine-Regulated Candidate Genes Predicts Poorer Glycemic Control
Source: J Diabetes Res. 2016 Jan 20;2016:9570424. doi: 10.1155/2016/9570424 (PMC4745814; doi:10.1155/2016/9570424)
Supplement: Supplementary file 1 — Supplementary Table 1: The risk allele distribution of the 11 T1D candidate genes. Supplementary Table 2.: Variance explained by regression models for HbA1c and IDAA1c with and without genetic risk score (GRS). [file 9570424.f1.docx]

Supplementary table 1. The risk allele distribution of the 11 T1D candidate genes.

| Risk allele distribution | Number of risk alleles |
| --- | --- |
| Min. | 7 |
| Max. | 18 |
| Median | 13 |
| 25th percentile | 12 |
| 75th percentile | 15 |

The risk allele distribution of the 11 T1D genes qualified by cytokine-induced human islet expression analysis (*GSDMB*, *TNFAIP3*, *COBL*, *CTRB1*, *SH2B3*, *IL7R*, *IFIH1*, *SKAP2*, *IL10*, *CTSH*, *INS*) among 182 children with new-onset T1D.

Supplementary table 2. Variance explained by regression models for HbA1c and IDAA1c with and without genetic risk score (GRS).

| Time after onset | R^2^ (variance explained by the model) | p-value (for model) | R^2^ (variance explained by the model) | p-value (for model) |
| --- | --- | --- | --- | --- |
| 1 month – no GRS | 0.13 | < 0.0001 | 0.11 | 0.0007 |
| 1 month – with GRS | 0.15 | < 0.0001 | 0.11 | 0.0008 |
| 3 months – no GRS | 0.04 | 0.15 | 0.05 | 0.12 |
| 3 months – with GRS | 0.08 | 0.02 | 0.06 | 0.12 |
| 6 months – no GRS | 0.02 | 0.8 | 0.05 | 0.16 |
| 6 months – with GRS | 0.08 | 0.03 | 0.07 | 0.06 |
| 9 months – no GRS | 0.03 | 0.4 | 0.05 | 0.16 |
| 9 months – with GRS | 0.07 | 0.09 | 0.08 | 0.02 |
| 12 months – no GRS | 0.05 | 0.12 | 0.07 | 0.03 |
| 12 months – with GRS | 0.09 | 0.02 | 0.10 | 0.006 |

All models included age, sex and HLA risk group.
